# Classical and alternate complement factor overexpression in non-obese weight matched women with polycystic ovary syndrome does not correlate with vitamin D

**DOI:** 10.3389/fendo.2022.935750

**Published:** 2022-12-21

**Authors:** Abu Saleh Md Moin, Thozhukat Sathyapalan, Alexandra E. Butler, Stephen L. Atkin

**Affiliations:** ^1^ Research Department, Royal College of Surgeons in Ireland Bahrain, Adliya, Bahrain; ^2^ Academic Endocrinology, Diabetes and Metabolism, Hull York Medical School, Hull, United Kingdom

**Keywords:** polycystic ovary syndrome, complement factors, C3, C4, vitamin D

## Abstract

**Introduction:**

Women with polycystic ovary syndrome (PCOS) exhibit complement factor expression changes that may be obesity-driven rather than an intrinsic facet of PCOS; furthermore, complement changes have been associated with vitamin D deficiency, a common feature of PCOS. Therefore, complement pathway proteins and vitamin D levels may be linked in PCOS.

**Methods:**

We measured plasma levels of complement pathway proteins by Slow Off-rate Modified Aptamer (SOMA)-scan plasma protein measurement for the classical (C4, C4a, and C4b) and alternative pathways (C3, C3b, iC3b, properdin, and factors B, D, and H) in weight and age-matched non-obese non-insulin resistant women with PCOS (n = 24) and control women (n = 24). Proteins that differed between groups were correlated with 25-hydroxyvitamin D_3_ (25(OH)D_3_) and 1,25-dihydroxyvitamin D_3_ (1,25(OH)_2_D_3_), measured by isotope-dilution liquid chromatography tandem mass spectrometry.

**Results:**

Women with PCOS had a higher free androgen index and anti-Mullerian hormone, though insulin resistance was comparable to controls; likewise, C-reactive protein, a marker of inflammation, was comparable between cohorts. In the alternative complement pathway, C3, iC3b, and properdin were increased in PCOS (p <0.05), while C4 in the classical pathway was increased (p <0.05). 25(OH)D_3_ levels positively correlated with C3b only in control subjects, with no correlation of 1,25(OH)_2_D_3_ with any of the proteins.

**Conclusion:**

In a non-obese PCOS population matched for age, insulin resistance and inflammation, initiating proteins of the classical and alternate complement cascades were increased. However, a positive correlation with 25(OH)D_3_ was only seen for C3b in control subjects, with no correlation to 1,25(OH)_2_D_3_, suggesting that the increase in complement proteins in PCOS is vitamin D-independent.

## Introduction

**Figure 1:**
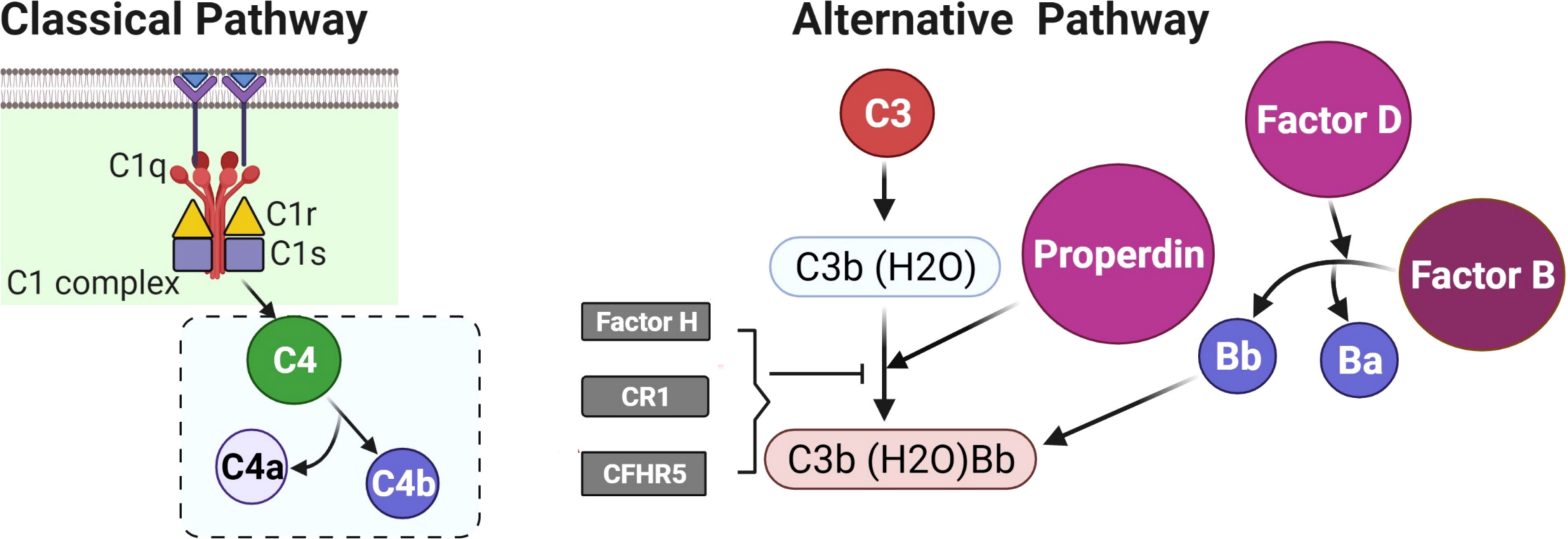
A schematic to illustrate the initiating proteins of the classical and alternate complement cascades.

In women with polycystic ovary syndrome (PCOS), there is an increased prevalence of type 2 diabetes, hypertension and, potentially, cardiovascular disease (1), the mechanism of which is still unclear, though inflammation has been implicated (2). Increased complement factor proteins in PCOS have been reported for both the classical and alternate cascade pathways, including C3, C4, properdin, factor B, and factor D (3) (**Figure 1**), though their expression and activation appeared to be dependent upon obesity and insulin resistance (3). Complement protein studies in PCOS have, however, been discrepant in the literature, with a confirmatory report that C3 may be elevated and related to inflammation (4) while, conversely, others report that C3 levels are unchanged (5).

Vitamin D deficiency is very common in women with PCOS, with 67%–85% being severely deficient, and low levels have been reported to correlate with obesity, insulin resistance, and testosterone levels (6, 7); however, it has been suggested that vitamin D does not exacerbate these features in PCOS (8). A systematic review and meta-analysis suggested that vitamin D deficiency was associated with insulin resistance but the significance was lost when BMI was accounted for (9). However, it has been reported that vitamin D insufficiency predicts elevated C3 levels independent of insulin resistance and obesity (10) and therefore, hypothetically, may contribute to the elevation of these proteins in PCOS. Vitamin D_3_ (cholecalciferol) is endogenously produced in the skin through the effect of UV-B on 7-dehydrocholesterol that is hydroxylated at position 25 to 25-hydroxyvitamin D_3_ (25(OH)D_3_). 25(OH)D_3_ is transported to the kidney and converted to the active 1,25(OH)_2_D_3_ by 1-alpha hydroxylase (11). This study was undertaken in a non-obese PCOS population versus controls matched for BMI, insulin resistance, and inflammation to determine whether complement factor proteins were independently associated with PCOS and whether they may be modulated by vitamin D metabolites.

## Materials and methods

We determined plasma complement pathway protein levels in women with PCOS (n = 24) and control women (n = 24) attending the Hull IVF clinic (11). Control women were age and body mass index (BMI) matched women with PCOS. Demographic data for both control women and women with PCOS are shown in **Table 1** (11). The Rotterdam consensus was used for the diagnosis of PCOS; these criteria are (1) clinical and biochemical hyperandrogenemia, requiring a Ferriman–Gallwey score of >8 and a free androgen index of >4, respectively (2), oligomenorrhea or amenorrhea and (3) polycystic ovaries seen on transvaginal ultrasound (12). Study participants had no other condition or illness and were required to be medication-free for nine months preceding study enrollment. Testing was undertaken to ensure that no patient had any of the following endocrine conditions: non-classical 21-hydroxylase deficiency, hyperprolactinemia, Cushing’s disease, or an androgen-secreting tumor. All procedures performed in studies involving human participants were in accordance with the ethical standards of the Yorkshire and The Humber NRES ethical committee, UK. In addition, they were in accordance with the 1964 Helsinki declaration and its later amendments or comparable ethical standards.

**Table 1:** Demographics, baseline, hormonal, and metabolic parameters of the women with PCOS and control women (mean ± SD). All parameters did not differ other than those marked **=p <0.01.

	Control (n = 24)	PCOS (n = 24)
Age (years)	32.5 ± 4.1	31 ± 6.4
BMI (kg/m^2^)	24.8 ± 1.1	25.9 ± 1.8
Fasting glucose (mmol/L)	4.9 ± 0.4	4.7 ± 0.8
HbA1C (mmol/mol)	30.9 ± 6.5	31.8 ± 3.0
HOMA-IR	1.8 ± 1.0	1.9 ± 1.6
SHBG (nmol/L)	104.2 ± 80.3	71.7 ± 62.2
Free androgen index (FAI)	1.3 ± 0.5	4.1 ± 2.9**
CRP (mg L^−1^)	2.34 ± 2.34	2.77 ± 2.57
AMH (ng/ml)	24 ± 13	57 ± 14**
25 hydroxy vitamin D3 (nmol/l)	46.2 ± 23.5	54.0 ± 27.4
1,25 Dihydroxy vitamin D3 (ng/ml)	0.03 ± 0.02	0.04 ± 0.2

BMI, Body Mass Index; HbA1c, glycated hemoglobin; HOMA-IR, Homeostasis model of assessment-insulin resistance; CRP, C reactive protein; SHBG, sex hormone binding globulin; AMH, Anti-Müllerian hormone.

Fasting blood samples were centrifuged at 3,500*g* for 15 min and placed into aliquots and frozen at −80°C until analysis. The blood samples were analyzed for sex hormone binding globulin (SHBG), insulin (DPC Immulite 200 analyzer, Euro/DPC, Llanberis UK), and plasma glucose (Synchron LX20 analyzer, Beckman-Coulter, High Wycombe, UK). Free androgen index (FAI) was calculated by dividing the total testosterone by SHBG, and then multiplying by 100. Insulin resistance (IR) was calculated using the homeostasis model assessment (HOMA-IR). Serum vitamin D levels and testosterone were quantified using isotope-dilution liquid chromatography tandem mass spectrometry (LC-MS/MS) (11). Circulating levels of complement pathway proteins were determined by Slow Off-rate Modified Aptamer (SOMA)-scan plasma protein measurement (Somalogic, Boulder, CO, USA), the details of which have been previously reported (13). Normalization of raw intensities, hybridization, median signal, and calibration signal were performed based on the standard samples included on each plate, as previously described (14).

We measured plasma complement pathway protein levels for the alternative (C3, C3b, iC3b, properdin, factors B, D, and H) and classical pathways (C4, C4a, and C4b) (3) (**Table 2**).

**Table 2:** Complement dysregulation in non-obese non-insulin resistant women with PCOS.

Complement pathway proteins	PCOS	Control	p-value
Properdin	152,592 (42,743)	117,488 (50,041)	0.006
iC3b	7,148 (2,127)	5,991 (1,425)	0.02
C3	65,878 (26,872)	45,742 (18,189)	0.002
C3b	50,982 (28,296)	46,250 (39,450)	0.6
C4	119,057 (28,429)	104,245 (28,069)	0.05
C4a	71,549 (9,802)	73,037 (2,258)	0.43
C4b	349 (186)	335 (202)	0.78
Factor D	733 (99)	693 (150)	0.24
Factor B	30,257 (6,541)	28,172 (5,791)	0.2
Factor H	60,898 (9,191)	59,289 (6,016)	0.43

Complement proteins for the classical (C4, C4a, and C4b) and alternative (C3, iC3, Properdin, Factors B, D, and H) complement pathways in non-obese non-insulin resistant women with polycystic ovary syndrome (PCOS) versus their BMI-matched controls.

Data presented as Mean ± 1 Standard Deviation of Relative Fluorescent Units (RFU).

### Statistics

**Figure 2:**
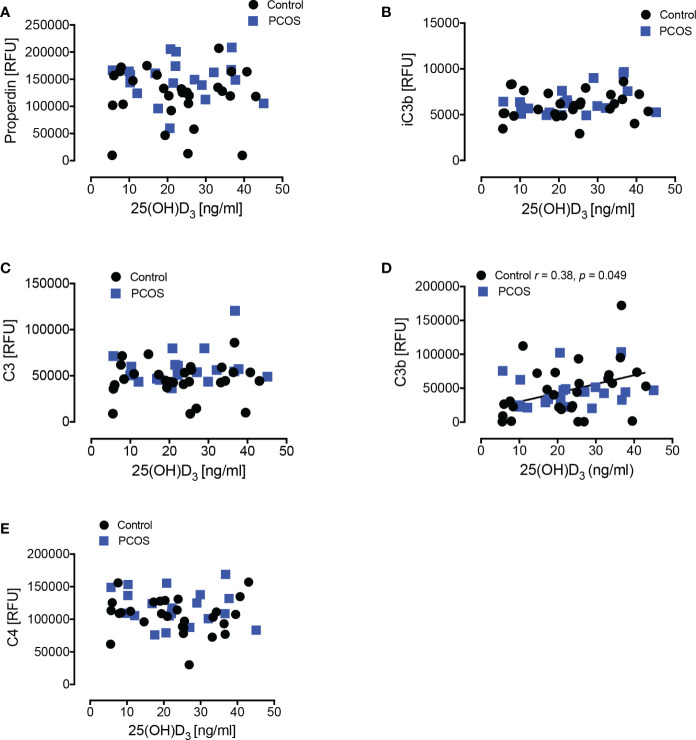
Correlations of complement pathway proteins with 25-hydroxy vitamin D_3_ [25(OH)D_3_]. Correlations of 25(OH)D_3_ with properdin **(A)**, iC3b **(B)**, C3 **(C)**, C3b **(D)**, and C4 **(E)** in women with PCOS and control women are shown. Only in controls did C3b show a positive correlation with 25(OH)D_3._ RFU, Relative Fluorescent Units.

A power analysis (nQuery version 9, Statsol USA) was undertaken for C3 protein that had been previously reported (3). For 80% power and an alpha of 0.05 with a common standard deviation of 0.37, the number of subjects required was 23. Data trends were visually and statistically evaluated for normality. Independent t-tests were applied to normally distributed data, while non-parametric tests (Mann–Whitney U) were applied to data that violated the assumptions of normality when tested using the Kolmogorov–Smirnov Test. Correlations between vitamin D and the differing complement proteins were undertaken with the Pearson coefficient and shown in **Figure 2** and **Supplementary Figure 1**. All analyses were performed using R version 4.0.0 (R Foundation for Statistical Computing, Vienna, Austria. URL: https://www.R-project.org/).

## Results

Baseline data for the 24 non-obese women with PCOS matched for age and BMI with 24 control subjects is shown in **Table 1**. Insulin resistance and CRP (as a marker of inflammation) did not differ between the two groups. Women with PCOS had an elevated free androgen index and an elevated anti-Mullerian hormone, as expected in PCOS.

The results of the complement factors are shown in **Table 2** for both the non-obese women with PCOS and the control women. In these non-obese, non-insulin-resistant women with PCOS, there were significant elevations in the levels of alternative pathway complement proteins C3 (p <0.002), iC3b (p <0.02), and properdin (p <0.006), while C4 in the classical pathway was increased (p <0.05); C4a and C4b did not differ between groups.

25(OH)D_3_ levels correlated with C3 for control subjects, but not for PCOS (**Figure 2**); however, there was no correlation for 1,25(OH)_2_D_3_ with any of the complement proteins in either cohort (**Supplementary Figure 1**).

## Discussion

These data show that, in non-obese women with PCOS that did not differ for insulin resistance and inflammation compared to controls, proteins in the alternative complement activation pathway (C3, iC3, and properdin) were elevated in women with PCOS in comparison with the matched controls; C4, belonging to the classical complement activation pathway, was also increased in PCOS. These data are in accord with those reported by others; of note, in that report, Factors B, D, and H were also elevated but became non-significant when the data was adjusted for BMI (3). C4 was elevated, but its products of activation (C4a and C4b) were no different between women with PCOS and controls, suggesting that activation of C4 was not occurring. Similarly, C3 was elevated, but its product of activation (C3b) was no different between women with PCOS and control women, again suggesting that activation of C3 was not taking place. However, properdin was elevated and, as its action is to stabilize C3 convertase in the alternative pathway, its elevation would prolong complement activation (15).

In this cohort of women with PCOS who were not obese, insulin resistant or demonstrating indices of inflammation greater than control women, this suggests that the alterations of the complement proteins in this study are reflective of the inherent processes in PCOS rather than an epiphenomenon reflective of obesity, insulin resistance or inflammation, as has been previously suggested by mathematical modeling (3). This is important, as many of the cardiovascular risk factors are largely due to obesity and its associated complications rather than the underlying pathophysiology of PCOS. It can, however, be difficult to determine the relative contributions of obesity and insulin resistance to the inherent dysfunction of PCOS (16, 17). Further adding to the complexity, the underlying disease process of PCOS may also be affected by ethnicity (18). The complement protein results reported here are in accord with some studies in PCOS (3, 4) but not others (5); however, those studies had not taken into account the underlying pathophysiology of obesity, insulin resistance and inflammation that are addressed here.

Vitamin D_3_ levels correlated positively with C3b in controls only, indicating that vitamin D_3_ deficiency was associated with lower C3b protein levels. However, no correlation was seen for the active form, 1,25(OH)_2_D_3._ A correlation between vitamin D and C3 was reported to be independent of insulin resistance and BMI in normal controls (10) though, in that study, C3b was not measured specifically. The lack of correlation seen for the active 1,25(OH)_2_D_3_ with any of the complement proteins suggests that the 25(OH)D_3_ association seen with C3b alone, together with the fact that C3b plasma protein levels did not differ between groups, may be a chance observation or an epiphenomenon secondary to some unknown factor in the control women. In either case, the data suggest that the changes in complement proteins seen in PCOS are not due to vitamin D levels.

### Limitations

A major concern of any study such as this that describes negative findings is that of reporting a type 2 statistical error due to an inadequate sample size. The initial power analysis was based on that for C3, and significant findings for iC3b, properdin and C4 were found in accord with others (3). The results for Factors B, D, and H are in accord with the modeling that they would become non-significant when BMI is considered and, therefore, in a non-obese population would not differ. As all study subjects were Caucasian, these results may not be generalizable to other ethnic populations. It would be important for future studies to address the role of the PCOS phenotype (19) in the presence and absence of obesity.

In conclusion, in a non-obese PCOS population matched for age, insulin resistance, and inflammation, initiating proteins of the classical and alternate complement cascades were increased in women with PCOS, but their positive correlation with 25(OH)D_3_ was only seen for C3b in control women, with no correlation to 1,25(OH)_2_D_3,_ suggesting that the increase in complement proteins in women with PCOS is independent of vitamin D.

## Data availability statement

The raw data supporting the conclusions of this article will be made available by the authors, without undue reservation.

## Ethics statement

The studies involving human participants were reviewed and approved by the Yorkshire and The Humber NRES ethical committee, UK. The patients/participants provided their written informed consent to participate in this study.

## Author contributions

AM and AB analyzed the data and wrote the manuscript. TS supervised clinical studies and edited the manuscript. SA contributed to study design, data interpretation and the writing of the manuscript. AB is the guarantor of this work. All authors contributed to the article and approved the submitted version.

## Supplementary material

Supplementary Figure 1 Correlations of complement pathway proteins with 1,25-dihydroxy vitamin D_3_ [1,25(OH)_2_D_3_]. No correlation of 1,25(OH)_2_D_3_ was seen with properdin **(A)**, iC3b **(B)**, C3 **(C)**, C3b **(D)** or C4 **(E)** in women with PCOS or control women. Relative Fluorescent Units (RFU).
