# Supplementary figures and images for: Classical and alternate complement factor overexpression in non-obese weight matched women with polycystic ovary syndrome does not correlate with vitamin D
Source: Front Endocrinol (Lausanne). 2022 Dec 21;13:935750. doi: 10.3389/fendo.2022.935750 (PMC9811138; doi:10.3389/fendo.2022.935750)

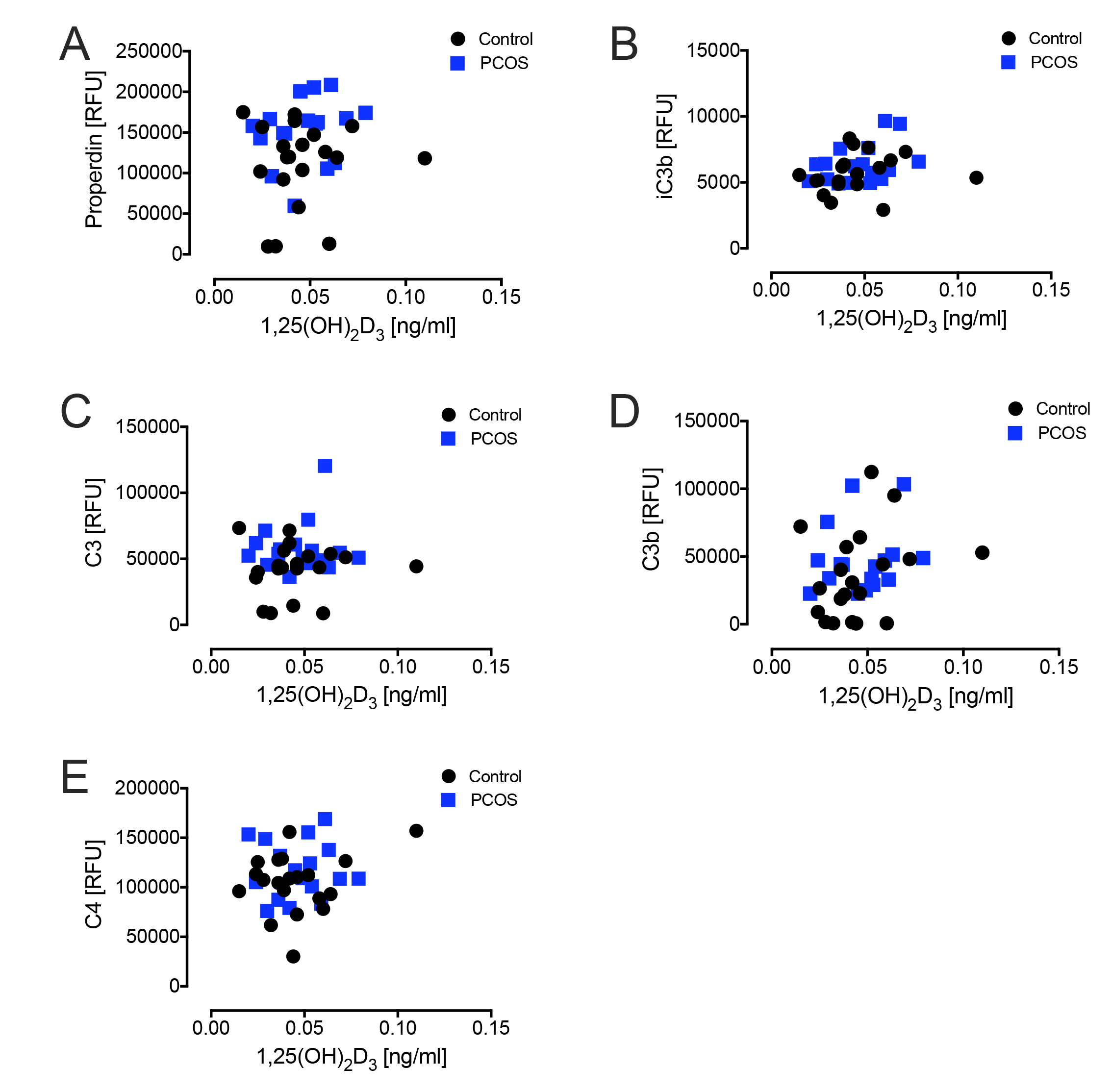

Supplement: Supplementary Figure 1 — Correlations of complement pathway proteins with 1,25-dihydroxy vitamin D3 [1,25(OH)2D3]. No correlation of 1,25(OH)2D3 was seen with properdin (A), iC3b (B), C3 (C), C3b (D) or C4 (E) in women with PCOS or control women. Relative Fluorescent Units (RFU). [file Image_1.tif]
